# *Arabidopsis* AtERF15 positively regulates immunity against *Pseudomonas syringae* pv. *tomato* DC3000 and *Botrytis cinerea*

**DOI:** 10.3389/fpls.2015.00686

**Published:** 2015-09-04

**Authors:** Huijuan Zhang, Lei Huang, Yi Dai, Shixia Liu, Yongbo Hong, Limei Tian, Lihong Huang, Zhongye Cao, Dayong Li, Fengming Song

**Affiliations:** National Key Laboratory for Rice Biology, Institute of Biotechnology, Zhejiang UniversityHangzhou, China

**Keywords:** *Arabidopsis thaliana*, ethylene-responsive factor (ERF) transcription factors, immune response, pattern-triggered immunity, *Pseudomonas syringae* pv. *tomato* DC3000, *Botrytis cinerea*

## Abstract

Upon pathogen infection, activation of immune response requires effective transcriptional reprogramming that regulates inducible expression of a large set of defense genes. A number of ethylene-responsive factor transcription factors have been shown to play critical roles in regulating immune responses in plants. In the present study, we explored the functions of *Arabidopsis* AtERF15 in immune responses against *Pseudomonas syringae* pv. *tomato* (*Pst*) DC3000, a (hemi)biotrophic bacterial pathogen, and *Botrytis cinerea*, a necrotrophic fungal pathogen. Expression of *AtERF15* was induced by infection of *Pst* DC3000 and *B. cinerea* and by treatments with salicylic acid (SA) and methyl jasmonate. Biochemical assays demonstrated that AtERF15 is a nucleus-localized transcription activator. The *AtERF15*-overexpressing (AtERF15-OE) plants displayed enhanced resistance while the AtERF15-RNAi plants exhibited decreased resistance against *Pst* DC3000 and *B. cinerea*. Meanwhile, *Pst* DC3000- or *B. cinerea*-induced expression of defense genes was upregulated in AtERF15-OE plants but downregulated in AtERF15-RNAi plants, as compared to the expression in wild type plants. In response to infection with *B. cinerea*, the AtERF15-OE plants accumulated less reactive oxygen species (ROS) while the AtERF15-RNAi plants accumulated more ROS. The flg22- and chitin-induced oxidative burst was abolished and expression levels of the pattern-triggered immunity-responsive genes *AtFRK1* and *AtWRKY53* were suppressed in AtER15-RNAi plants upon treatment with flg22 or chitin. Furthermore, SA-induced defense response was also partially impaired in the AtERF15-RNAi plants. These data demonstrate that AtERF15 is a positive regulator of multiple layers of the immune responses in *Arabidopsis*.

## Introduction

Plants have evolved to possess a sophisticated innate immune system to defend themselves against pathogen attack during their lifespan (Boller and He, 2009; Dodds and Rathjen, 2010; Schwessinger and Ronald, 2012; Fu and Dong, 2013). To date, two types of innate immune responses that are timely activated and precisely regulated by different types of pathogens have been recognized in plants and studied extensively for their molecular, genetic and biochemical mechanisms. Pathogen-associated molecular pattern (PAMP)-triggered immunity (PTI) is often activated by PAMPs such as flagellin, EF-Tu and chitin, most of which are common structural components of microbes (Zhang and Zhou, 2010; Bernoux et al., 2011; Segonzac and Zipfel, 2011); whereas effector-triggered immunity is modulated by recognition of pathogen-derived specific avirulence effectors by plant R proteins (Cui et al., 2009; Bigeard et al., 2015). In addition to the innate immunity, plants have also developed to possess several forms of inducible immunity that becomes activated upon pathogen infection or treatment of elicitors. Systemic acquired resistance (SAR) and induced systemic resistance (ISR), are the two types of inducible immunity that is activated by different stimuli through distinct signaling pathways (Grant and Lamb, 2006; Van Wees et al., 2008; Shoresh et al., 2010; Fu and Dong, 2013). Once the pathogen-derived PAMPs or effectors and elicitors are perceived (Boller and He, 2009), a battery of immune responses is often activated by a network of defense hormone-mediated signaling pathways (Pieterse et al., 2009), which ultimately lead to transcriptional reprogramming that coordinately regulates expression of a large set of genes. The transcription reprogramming is the consequence of the concerted action of a range of transcription factors (TFs), which function directly or indirectly to deploy their activity in proper ways. TFs are divided into diverse families according to conserved structural domains, which are involved in DNA binding activity and numerous TFs belonging to the AP2/ERF, NAC, WRKY, and bZIP families have been implicated in plant immune responses against diverse pathogens (Gutterson and Reuber, 2004; Alves et al., 2013; Nuruzzaman et al., 2013; Seo et al., 2015).

The AP2/ERF superfamily is a large plant-specific TF family and consists of approximately 147 members in the *Arabidopsis*, of which 122 are the ERF proteins (Sakuma et al., 2002; Gutterson and Reuber, 2004; Nakano et al., 2006). The ERF proteins contain a highly conserved DNA-binding domain, which is consisted of 58 or 59 amino acid residues (Ohme-Takagi and Shinshi, 1995). Many of them have been shown to bind specifically to GCC box, a core sequence essential for activation of expression of defense genes (Ohme-Takagi and Shinshi, 1995; Büttner and Singh, 1997; Zhou et al., 1997; Solano et al., 1998). The ERFs can act as transcription activators or repressors in regulating the expression of defense genes. For example, the *Arabidopsis* AtERF1, AtERF2, and AtERF5 are activators while AtERF3, AtERF4, and AtERF7 act as repressors (Fujimoto et al., 2000).

The 122 *Arabidopsis* AtERF proteins have been divided into 12 major groups on the basis of the type of AP2 domain (Nakano et al., 2006) and many of them have not been defined for their biological functions yet. Recent studies using loss-of-function and gain-of-function *Arabidopsis* mutants have demonstrated that a number of the ERFs in B3 group, consisting of 17 members, play critical roles in regulating defense responses against pathogens. For example, it was shown that *AtERF92* (*AtERF1*), *AtERF100* (*AtERF-1*), *AtERF101* (*AtERF2*), *AtERF97* (*AtERF14*) and *AtERF94* (*ORA59*) are responsive transcriptionally to pathogens and defense signaling molecules such as salicylic acid (SA), jasmonic acid (JA), and ethylene (ET) (Oñate-Sánchez and Singh, 2002; McGrath et al., 2005; Oñate-Sánchez et al., 2007; Pré et al., 2008). It was found that overexpression (OE) of *AtERF1*, *AtERF2*, *AtERF5*, *AtERF6*, *AtERF14*, or *ORA59* conferred an increased resistance against several pathogenic fungi including *Botrytis cinerea* through activating the expression of defense-related genes such as *AtPDF1.2* and *ChiB* (Solano et al., 1998; Berrocal-Lobo et al., 2002; Brown et al., 2003; Lorenzo et al., 2003; McGrath et al., 2005; Oñate-Sánchez et al., 2007; Pré et al., 2008; Moffat et al., 2012). The functions of ERF1, AtERF5, AtERF6, AtERF14, and ORA59 have been shown to act as regulators of the JA/ET-mediated signaling pathway (Lorenzo et al., 2003; Berrocal-Lobo and Molina, 2004; Oñate-Sánchez et al., 2007; Pré et al., 2008; Moffat et al., 2012). More recently, it was found that phosphorylation of AtERF6 by MPK3/MPK6 can increase the AtERF6 stability and thus constitutively activates defense genes including *AtPDF1.1* and *AtPDF1.2* and provides increased resistance against *B. cinerea* (Meng et al., 2013). In addition, AtERF5 was shown to be involved in the chitin-induced innate immunity response (Son et al., 2012). Therefore, 7 out of 17 B3 group members in the ERF family play important roles in regulating defense response against pathogens. However, the function of the other B3 group members in plant defense response remains unknown.

AtERF15, a member of the B3 group in the AtERF family, was recently shown to function as a positive regulator of abscisic acid (ABA)-mediated abiotic stress response (Lee et al., 2015). In this study, we explored the function of AtERF15 in immune response against *Pseudomonas syringae* pv. *tomato* (*Pst*) DC3000 and *B. cinerea* by analyzing the disease phenotypes and defense response in AtERF15-overexpressing (AtERF15-OE) and AtERF15-RNAi lines. Our data revealed that constitutive OE of *AtERF15* conferred an increased resistance while RNAi-mediated suppression of *AtERF15* led to decreased resistance against *Pst* DC3000 and *B. cinerea*. Furthermore, suppression of *AtERF15* attenuated the flg22- or chitin-induced PTI response and partially impaired the SA-induced resistance. Our data demonstrate that AtERF15 is a positive regulator of multiple layers of the immune responses in *Arabidopsis*.

## Materials and Methods

### Plant Growth and Treatment

*Arabidopsis thaliana* ecotype Col-0 and transgenic plants were cultivated in a mixture of vermiculite: plant ash: perlite (6:2:1) in a growth room with a rhythm of 16 h light/8 h dark. For treatments with signaling hormones, 1 mM SA and 100 μM methyl jasmonate (MeJA) in 0.1% ethanol were sprayed evenly on the leaves of 4-week-old plants. Mock controls were set by spraying a group of plants with similar volume of 0.1% ethanol.

### Generation and Characterization of Transgenic Lines

The open reading frame (ORF) of *AtERF15* was obtained by RT-PCR using a pair of gene-specific primers AtERF15-orf-1F (5′-ATG GAA TAT TCC CAA TCT-3′) and AtERF15-orf-1R (5′-TCA ACA TGA GCT CAT AAG-3′) and cloned into pMD19-T vector, yielding plasmid pMD19-AtERF15. After confirmation by sequencing, plasmid pMD19-AtERF15 was used for all experiments. To construct the OE plasmid, the *AtERF15* ORF was amplified from pMD19-AtERF15 using a pair of primers AtERF15-orf-2F (5′-AGT GGA TCC ATG GAA TAT TCC CAA TCT-3′, a *Bam*HI site underlined) and AtERF15-orf-2R (5′-AGA GGG CCC TCA ACA TGA GCT CAT AAG -3′, a *Apa*I site underlined), and cloned into binary vector pCAMBIA 99-1 under control of the CaMV 35S promoter, yielding pCAMBIA991-AtERF15. To construct the *AtERF15* RNAi plasmid, a fragment of 237 bp in size was amplified from pMD19-AtERF15 using a pair of primers AtERF15-Ri-1F (5′-AGA TTAATTAA CCATGG GAT AAC AAA AAG AAA AGA AAA AGA G-3′, *Pac*I/*Nco*I sites underlined) and AtERF15-Ri-1R (5′-GCG GGATCC GGCGCGCC TAA TCA TCA TCT CCG GTG ACT CAA A-3′, *Bam*HI/*Asc*I sites underlined) and cloned into vector pGSA1165 under control of the CaMV 35S promoter, pGSA1165-AtERF15. *Arabidopsis* transformation was carried out by the floral dip method (Clough and Bent, 1998). Single-copy homozygous lines were selected according to the segregation ratios (hygromycin-rsesistant:hygromycin-sensitive = 3:1) on selective 1/2 MS medium containing 50 μg/ml hygromycin.

### Pathogen Inoculation and Disease Assays

*Pseudomonas syringae pv. tomato* DC3000 was cultivated in King’s B (KB) liquid medium with 25 μg/ml rifampicin at 28°C in a shaker to OD_600_ = 0.7∼1.0. Bacterial inoculum was prepared in 10 mM MgCl_2_ and adjusted to OD_600_ = 0.002. Inoculation was done using 1-ml syringes without needles by infiltrating bacterial suspension into the leaf blade or by infiltrating with similar volume of 10 mM MgCl_2_ solution as mock-inoculation controls. The inoculated plants were covered with a transparent plastic film to keep high humidity and photos were taken 4 days after inoculation. For the determination of bacteria population, leaf disks from six independent plants were first sterilized with 70% ethanol for 10 s, homogenized in 200 μl 10 mM MgCl_2_ and diluted to the proper concentration and plated on KB agar plates supplemented with 100 μg/ml rifampicin.

*Botrytis cinerea* strain BO5.10 (provided by Dr. Tesfaye Mengiste, Purdue University, USA) (Veronese et al., 2006) was cultivated on 2 × V8 agar medium (36% V8 juice, 0.2% CaCO_3_, and 2% agar) for 10 days. Spores were collected in 1% maltose buffer to prepare the inoculum and adjusted to a final concentration of 2 × 10^5^ spores/ml. Inoculation was carried out by spraying the spore suspension until the leaves were covered with tiny moisture (Wang et al., 2009). Chlorophyll contents were measured as described previously (Veronese et al., 2006; Wang et al., 2009) and shown as percentages of that in the corresponding mock-inoculated plants. The completely rotten plants were recorded as died plants. *In planta* fungal growth was estimated by analyzing the transcript levels of *B. cinerea BcActinA* gene using a pair of primers BcActinA-1F (5′-ACT CAT ATG TTG GAG ATG AAG CGC AA-3′) and BcActinA-1R (5′-AAT GTT ACC ATA CAA ATC CTT ACG GAC A-3′). The *Arabidopsis* actin gene (*AtActin*) was used as an internal control and the primers were AtActin-1F (5′-GGC GAT GAA GCT CAA TCC AAA CG-3′) and AtActin-1R (5′-GGT CAC GAC CAG CAA GAT CAA GAC G-3′).

### Subcellular Localization Assays

The *AtERF15* ORF was amplified from pMD19-AtERF15 using a pair of gene-specific primers AtERF15-s-1F (5′-GCG TCTAGA ATG GAA TAT TCC CAA TCT-3′, a *Xba*I site underlined) and AtERF15-s-1R (5′-ATA CCCGGG TCA ACA TGA GCT CAT AAG-3′, a *Sma*I site underlined) and inserted into vector pFGC-EGFP, yielding plasmid pFGC-GFP-AtERF15. This plasmid and the pFGC-EGFP empty vector were electroporated into *Agrobacterium tumefacies* GV3101 and the acquired agrobacteria were injected into leaves of 4-week-old *Nicotiana benthamiana* plants expressing a red nuclear marker protein RFP–H2B (Chakrabarty et al., 2007) using 1-ml needless syringes. After agroinfiltration, the plants were grown in a growth room under 25°C for 24 h. GFP fluorescence signals were excited at 488 nm and detected using a 500–530 nm emission filter preformed with confocal laser scanning microscope (Zeiss LSM 510 META).

### Transactivation Activity Assays

The *AtERF15* ORF was amplified from pMD19-AtERF15 using a pair of gene-specific primers AtERF15-y-1F (5′-AGT GTCGAC ATG GAA TAT TCC CAA TCT-3′, a *Sal*I site underlined) and AtERF15-y-1R (5′-GCG CTGCAG TCA ACA TGA GCT CAT AAG-3′, a *Pst*I site underlined) and constructed into vector pBD-GAL4Cam, yielding plasmid pBD-AtERF15. Plasmid pBD-AtERF15, pBD-GAL4 (a positive control) and pBD empty vector (a negative control) were transformed into yeast strain AH109. The transformed yeasts were plated on deficient medium (*SD*/Trp^-^ His^-^) with x-α-gal and incubated for 3 days at 30°C. Transactivation activity of the fused proteins was evaluated based on the emergence of blue pigments.

### RNA Extraction and qRT-PCR Analysis of Gene Expression

Total RNA was extracted by Trizol regent (TaKaRa, Dalian, China) following the manufacturer’s instructions and the first-strand cDNA was obtained using PrimeScript RT regent kit (TaKaRa, Dalian, China). The qPCR reaction was done on a CFX96 real-time PCR system (BioRad, Hercules, CA, USA) in 25 μL reactions containing 12.5 μL SYBR Premix Ex TaqTM (TaKaRa, Dalian, China), 0.1 mg cDNA and 7.5 pmol of each gene-specific primer. Relative gene expression levels were calculated using 2^-ΔΔCT^ method with three independent biological replicates. Primers used in qRT-PCR were AtPR1-q-F, 5′-TCG TCT TTG TAG CTC TTG TAG GTG-3′; AtPR1-q-R, 5′-TAG ATT CTC GTA ATC TCA GCT CT-3′; AtPR5-q-F, 5′-ATG GCA AAT ATC TCC AGT ATT CAC A-3′; AtPR5-q-R, 5′-ATG TCG GGG CAA GCC GCG TTG AGG-3′; AtPDF1.2-q-F, 5′-GCTA AGT TTG CTT CCA TCA TCA CCC TT-3′; AtPDF1.2-q-R, 5′-AAC ATG GGA CGT AAC AGA TAC ACTTGT G-3′; AtActin-q-1F, 5′-GGC GAT GAA GCT CAA TCC AAA CG-3′; AtActin-q-1R, 5′-GGT CAC GAC CAG CAA GAT CAA GAC G-3′. AtNPR1-q-1F, 5′-CAC TAT GGC GGT TGA ATG TA-3′; AtNPR1-q-1R, 5′-GGG AGG AAC ATC TCT AGG AA-3′; AtERF15-q-1F, 5′-AAC GGC GAC GTT TCT AAC TCC GAA-3′; AtERF15-q-1R, 5′-GCT TTG TCA AAT GTC CCG AGC CAA-3′; AtFRK1-F, 5′-GCC AAC GGA GAC ATT AGA G-3′; AtFRK1-R, 5′-CCA TAA CGA CCT GAC TCA TC-3′; AtWRKY53-F, 5′-CAC CAG AGT CAA ACC AGC CAT TAC-3′; AtWRKY53-R, 5′-CTT TAC CAT CAT CAA GCC CAT CGG-3′.

### *In situ* Detection of ROS and Measurement of Oxidative Burst

*In situ* detection of superoxide anion and H_2_O_2_ was carried out using nitroblue tetrazolium (NBT) staining (Doke, 1983) and 3, 3-diaminobenzidine (DAB) staining (Thordal-Christensen et al., 1997; Li et al., 2014), respectively. Accumulation of superoxide anion and H_2_O_2_ in stained leaves was recorded by a digital camera. Determination of oxidative burst in leaves was carried out using a luminol-based luminescence method (Chakravarthy et al., 2010). Simply, 4-mm leaf disks from fully expanded leaves of 4-week-old plants were floated in 200 μL ddH_2_O overnight in each well of a 96-well plate, followed by addition of 100 nM flg22 (VI-A, Sigma, St. Louis, MO, USA) or 1 μM chitin (Hepta-*N*-acetylchitoheptaose, Toronto Research Chemicals, Toronto, ON, Canada), 34 μg/ml luminol (Sigma, St. Louis, MO, USA) and 20 μg/ml horseradish peroxidase (VI-A, Sigma, St. Louis, MO, USA). Addition of the same solutions without flg22 or chitin was used as controls. Luminescence was measured on a Synergy HT plate reader (Biotek Instruments, Inc. Winooski, VT, USA) and recorded every 2 min until 20 min.

### Identifiers of the Genes Used in This Study

*AtERF1*, At3g23240; *AtERF2*, At5g47220; *AtERF3*, At1g50640; *AtERF4*, At3g15210; *AtERF5*, At5g47230; *AtERF6*, At4g17490; *AtERF7*, At3g20310; *AtERF15*, At2g31230; *AtERF94* (*ORA59*), At1g06160; *AtERF97* (*AtERF14*), At1g04370; *AtFRK1*: At2g19190; *AtWRKY53*, At4g23810; *AtPR1*, At2g14610; *AtPR5*, At1g75040; *AtPDF1.2*, At5g4420; *AtNPR1*, At1g64280; *AtActin*, At1g01130.

## Results

### *AtERF15* was Induced by Infection with Pathogens and Treatments of SA and MeJA

**FIGURE 1:**
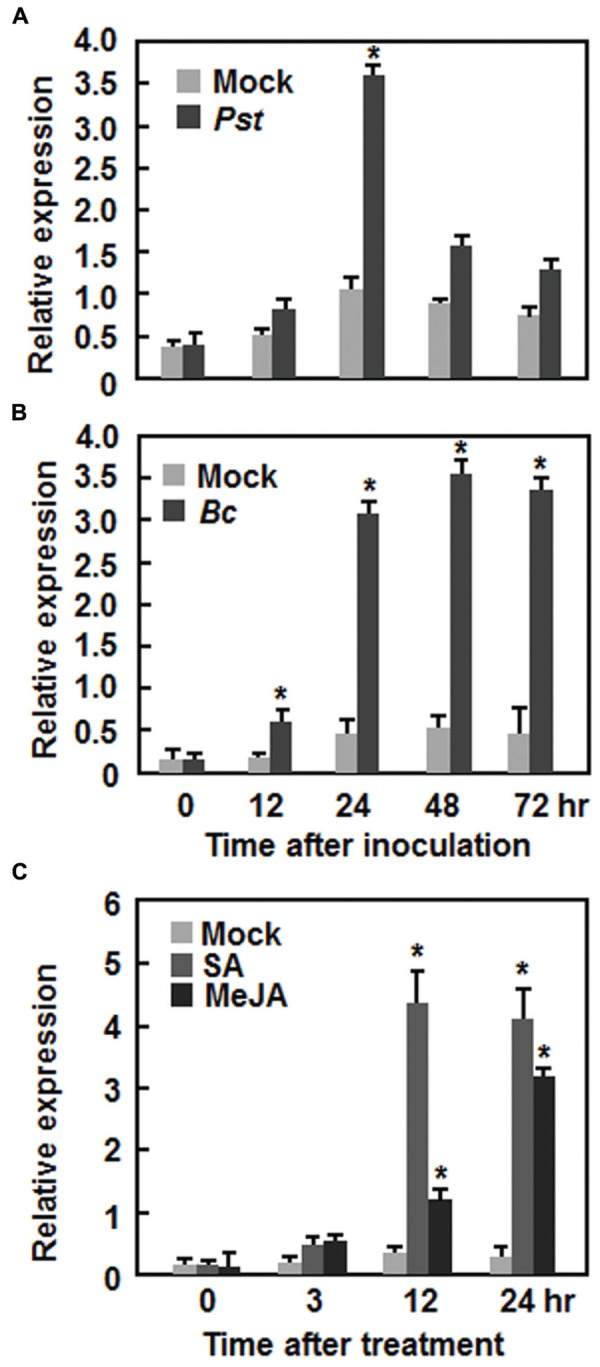
**Induction of *AtERF15* by infection with *Pst* DC3000 and *Botrytis cinerea* and by treatments with salicylic acid and methyl jasmonate. (A)** Induction of *AtERF15* by *Pst* DC3000. **(B)** Induction of *AtERF15* by *B. cinerea*. Four-week-old Col-0 plants were inoculated by vacuum infiltration with *Pst* DC3000 (OD_600_ = 0.002) **(A)**, by spraying with spores of *B. cinerea* (2 × 10^5^ spores/ml) **(B)**, or by treatment with corresponding solution (10 mM MgCl_2_) for *Pst* DC3000 and buffer (4% maltose and 1% peptone) for *B. cinerea* as mock controls. **(C)** Induction of *AtERF15* by SA and MeJA. Four-week-old Col-0 plants were treated by foliar spraying with 1 mM salicylic acid (SA), 100 μM jasmonic acid (MeJA), or 0.1% ethanol as mock controls. The inoculated or treated leaves were collected at indicated times and expression of *AtERF15* was analyzed through qRT-PCR. Data were normalized with the transcript level of *AtActin* as an internal control and relative expression levels were shown as folds of the level of *AtActin*. Data presented are the means ± *SD* from three independent experiments and ^∗^ above the columns indicate significant differences at *p* < 0.05 level between the inoculated/treated plants and mock control plants.

*AtERF15* encodes a 243 amino acid protein with high level of similarity to ORA59, which was shown to JA and ET signals in defense response (Pré et al., 2008), and belongs to Group IX (also known as B-3 group) of the *Arabidopsis* ERF family (Nakano et al., 2006). To explore its possible involvement in plant defense, we first analyzed the expression patterns of *AtERF15* in response to infection by different pathogens including *Pst* DC3000 and *B. cinerea* and to treatments with defense signaling hormones such as SA and MeJA. In *Pst* DC3000-infected plants, the expression of *AtERF15* was significantly induced at 24 h after inoculation, leading to a threefold increase over that in mock-inoculated plants, and then decreased (**Figure 1A**). Similarly, the expression of *AtERF15* in *B. cinerea*-infected plants was significantly induced at 12 h and maintained at relatively high levels during a period of 24–72 h after inoculation, giving 5 ∼ 6-fold increases over that in mock-inoculated plants (**Figure 1B**). Further, expression of *AtERF15* was upregulated by SA or JA but displayed distinct patterns. The expression of *AtERF15* in SA-treated plants increased significantly by 10-fold at 12 h and maintained at similar level at 24 h, as compared to mock control plants, after treatment (**Figure 1C**), whereas the expression of *AtERF15* in JA-treated plants increased by 2.3-fold at 12 h and further increased by 10-fold at 24 h, as compared to that in mock control plants, after treatment (**Figure 1C**). These results indicate that *AtERF15* is responsive to pathogens and defense signaling hormones, implying an involvement of *AtERF15* in defense response.

### AtERF15 is a Nucleus-Localized Protein with Transactivation Activity

**FIGURE 2:**
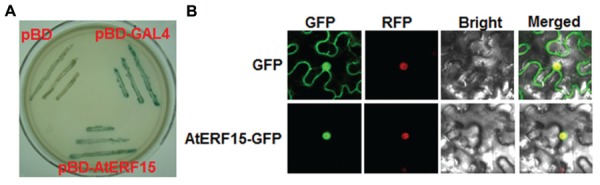
**Transactivation activity and nuclear localization of AtERF15 protein. (A)** AtERF15 has transactivation activity. Yeast cells carrying pBD-AtERF15, pBD-GAL4 (a positive control) or pBD empty vector (a negative control) were grown on *SD*/Trp^-^ plates for 3 days at 30°C and activity of β-galactosidase was detected by addition of x-α-gal. **(B)** AtERF15 is localized in nucleus. Agrobacteria carrying pFGC-Egfp-AtERF15 or pFGC-Egfp empty vector were infiltrated into leaves of *Nicotinana benthamiana* plants expressing a red nucleus marker protein RFP-H2B and leaf samples were collected at 24 h after infiltration for observation under a confocal laser scanning microscope. Images were taken in dark field for GFP and RFP, bright field for cell morphology and in combination, respectively.

The biochemical features of AtERF15 were examined by analyzing the transactivation activity in yeast and subcellular localization *in planta*. All yeasts transformed with pBD-AtERF15, pBD-Gal4 (a positive control) or pBD empty vector (a negative control) grew well on *SD*/Trp^-^ medium. However, only yeasts transformed with pBD-AtERF15 or pBD-Gal4 produced a blue pigment after the addition of x-α-gal, indicating a β-galactosidase activity, whereas yeasts transformed with empty vector did not (**Figure 2A**). To examine the subcellular localization, agrobacteria carrying pFGC-EGFP::AtERF15 and pFGC-EGFP (a negative control) were injected into leaves of 4-week-old *N. benthamiana* plants harboring a red nuclear marker protein RFP–H2B (Chakrabarty et al., 2007). The GFP::AtERF15 fusion was solely and clearly localized to the nucleus, co-localized with the known nucleus marker RFP–H2B protein (**Figure 2B**), whereas the GFP alone distributed ubiquitously throughout the cell without specific compartmental localization (**Figure 2B**). These data demonstrate that AtERF15 is a nucleus-localized protein with transactivation activity.

### Generation and Characterization of *AtERF15*-Overexpressing and RNAi Lines

**FIGURE 3:**
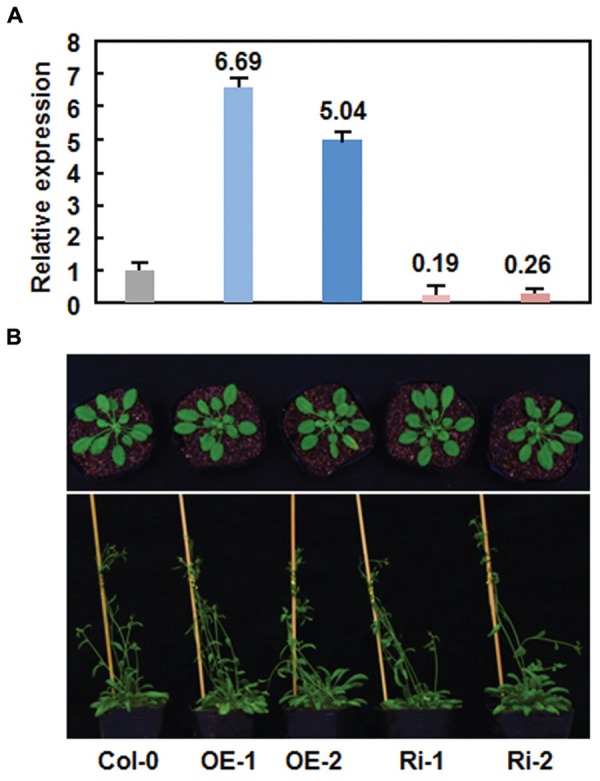
**No defects in growth and reproduction of the *AtERF15*-overexpressing (OE) and *AtERF15*-RNAi (Ri) plants. (A)** Expression levels of *AtERF15* in AtERF15-OE and AtERF15-Ri lines analyzed by qRT-PCR. **(B)** Morphological comparison of the *AtERF15*-OE and *AtERF15*-Ri plants with the Col-0 plants. Top panel, 4-week-old plants; bottom panel, 6-week-old plants.

Due to unavailability of appropriate T-DNA insertion mutant line for *AtERF15* gene at the beginning of this study, we thus generated CaMV 35S promoter-driven OE and RNA interfering (Ri)-mediated suppression lines to determine the biological function of AtERF15 in disease resistance. Transgenic AtERF15-OE and AtERF15-Ri lines were obtained through floral dip transformation and the single-copy homozygous T3 lines were obtained from T2 seeds according to the 3:1 (Hgr-resistant/Hgr-sensitive) segregation ratio. Two AtERF15-OE and two AtERF15-Ri lines were selected for further functional studies. qRT-PCR analysis revealed that the expression levels of *AtERF15* in AtERF15-OE lines were 6.69 and 5.04 times higher than that in Col-0 plants while the expression levels of *AtERF15* in AtERF15-Ri lines were 19 and 26% of the that in Col-0 plants (**Figure 3A**), indicating that the transgene is expressed correctly in both of the AtERF15-OE and AtERF15-Ri lines. In our experiment, no visible morphological or vegetable growth or reproductive development defect was observed in the AtERF15-OE and AtERF15-Ri plants (**Figure 3B**).

### Altered Disease Phenotypes and Defense Response in AtERF15-OE and AtERF15-Ri Plants Against *Pst* DC3000

**FIGURE 4:**
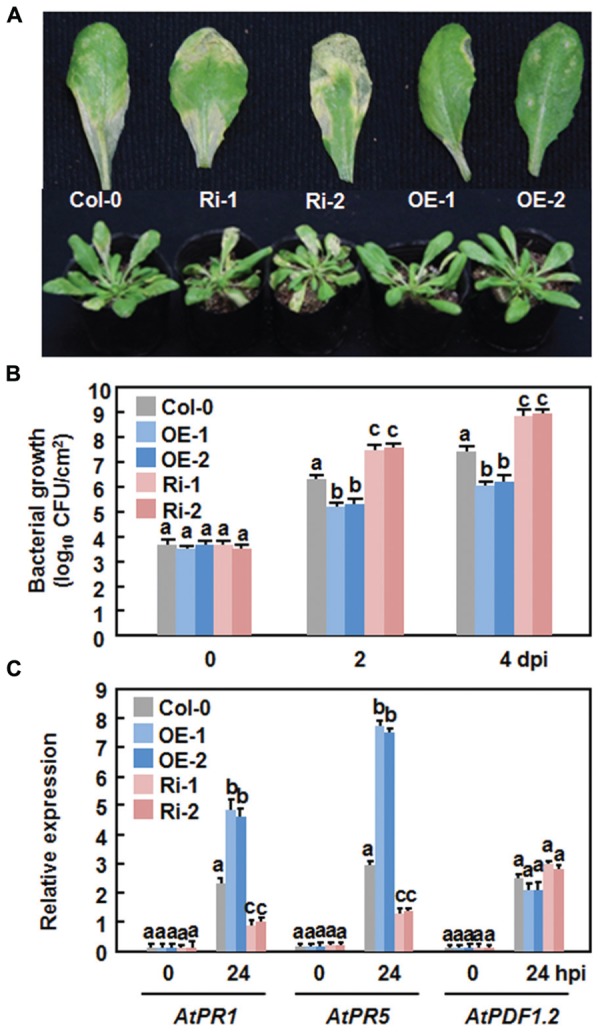
**Altered disease responses of the *AtERF15*-OE and *AtERF15*-Ri plants to *Pst* DC3000. (A)** Disease symptom on Col-0, *AtERF15*-OE and *AtERF15*-Ri plants. Four-week-old plants were infiltrated with *Pst* DC3000 (OD_600_ = 0.002). Photos were taken 4 days after inoculation. **(B)** Bacterial growth in infected leaves of Col-0, *AtERF15*-OE and *AtERF15*-Ri plants. Leaf samples were collected at 0, 2, and 4 days after inoculation and bacterial growth in CFU/cm^2^ leaf area was shown. **(C)** Expression of defense genes in Col-0, AtERF15-OE and AtERF15-Ri plants after inoculation with *Pst* DC3000. Expression of defense genes was analyzed by qRT-PCR and the transcript data obtained were normalized with the value of a reference *AtActin* gene. Relative expression levels of the defense genes were expressed as folds of the *AtActin* gene. Data presented are the means ±*SD* from three independent experiments and and different letters above the columns indicate significant differences at *p* < 0.05 level between the AtERF15-OE or AtERF15-Ri and Col-0 plants.

We first examined the function of AtERF15 in immunity against a bacterial pathogen by comparing disease phenotypes of the AtERF15-OE and AtERF15-Ri plants after challenging with a virulent strain of *Pst* DC3000. In the Col-0 plants, typical lesions were seen and bacterial growth in inoculated leaves was 1.6 × 10^6^ and 2.3 × 10^7^ CFU/cm^2^ at 2 and 4 days post-inoculation (dpi), respectively (**Figures 4A,B**). The AtERF15-OE plants showed less disease symptoms than the Col-0 plants after inoculation with the bacterial pathogen (**Figure 4A**). Similarly, bacterial growth in infected leaves of the AtERF15-OE-1 and AtERF15-OE-2 plants was (1.4 and 9.1) × 10^5^ and (1.6 and 11.5) × 10^5^ CFU/cm^2^ at 2 and 4 dpi, respectively, resulting in 10 ∼ 20 folds of decrease in bacterial growth relative to the Col-0 plants (**Figure 4B**). By contrast, the AtERF15-Ri plants developed more severe disease symptoms than the Col-0 plants, forming larger chlorotic lesions on the inoculated leaves (**Figure 4A**). The bacterial growth in inoculated leaves of the AtERF15-Ri-1 and AtERF15-Ri-2 plants was 2.9 × 10^7^ and 7.4 × 10^8^ CFU/cm^2^ and 4.1 × 10^7^ and 9.6 × 10^8^ CFU/cm^2^ at 2 and 4 dpi, respectively, resulting in significant increases of 18 ∼ 41 folds in bacterial growth, compared with those in Col-0 plants (**Figure 4B**). These results indicate that OE of AtERF15 resulted in a decreased *in planta* bacterial growth and less disease symptoms and while suppression of AtERF15 led to an increased *in planta* bacterial growth and severe disease symptoms.

To examine whether the altered disease phenotypes observed in AtERF15-OE and AtERF15-Ri plants are linked to changes in the regulation of defense responses due to OE or suppression of *AtERF15*, we measured the expression levels of some defense-related genes (i.e., *AtPR1*, *AtPR5*, and *AtPDF1.2*) in the Col-0,x AtERF15-OE and AtERF15-Ri plants after infection of *Pst* DC3000. Without infection, the expression levels of the selected defense genes in the AtERF15-OE and AtERF15-Ri plants were similar to those in the Col-0 plants (**Figure 4C**), indicating that altered expression of *AtERF15* did not affect the expression of defense-related genes. The expression of *AtPR1* and *AtPR5* in the Col-0 plant was significantly induced by *Pst* DC3000 at 24 h after inoculation, as compared with those in the mock-inoculated plants (**Figure 4C**). When compared with those in the Col-0 plants, the pathogen-induced expression of *AtPR1* and *AtPR5* showed 2- or 3-fold increase in the AtERF15-OE plants but had 2 or 3-fold reduction in the AtERF15-Ri plants at 24 h after inoculation with *Pst* DC3000 (**Figure 4C**). The expression of *AtPDF1.2* in the Col-0, AtERF15-OE and AtERF15-Ri plants was slightly affected in the same way by infection of *Pst* DC3000 (**Figure 4C**). These data indicate the OE or suppression of *AtERF15* can prime or attenuate the activation of defense response in *Arabidopsis* plants upon infection with *Pst* DC3000, linking to the altered immunity in the AtERF15-OE and AtEF15-Ri plants.

### Altered Disease Phenotypes and Defense Response in AtERF15-OE and AtERF15-Ri Plants Against *B. cinerea*

**FIGURE 5:**
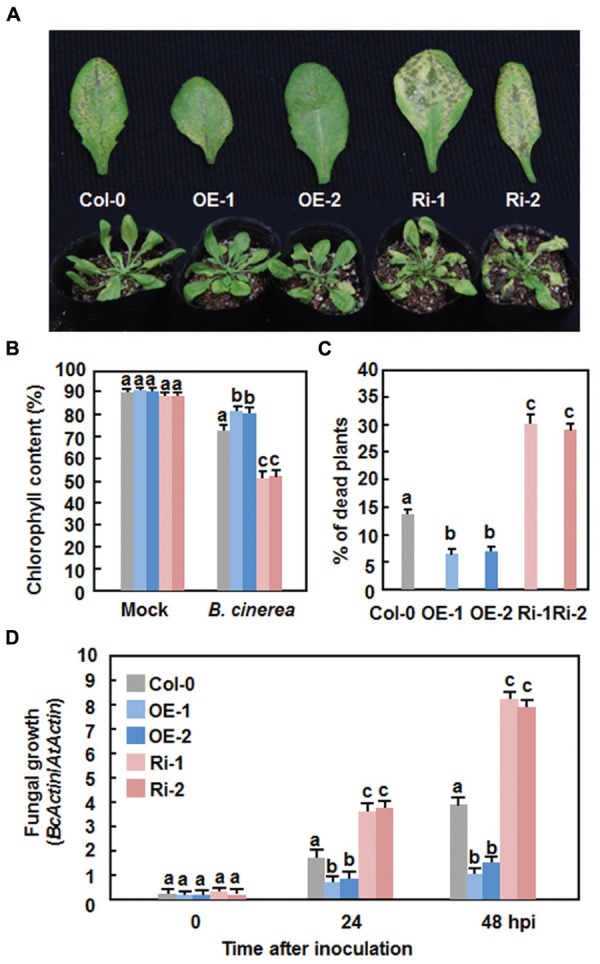
**Altered disease responses of *AtERF15*-OE and *AtERF15*-Ri plants to *B. cinerea*. (A)** Disease symptom on the *AtERF15*-OE and *AtERF15*-RNAi plants. Four-week-old plants were inoculated by foliar spraying with *B. cinerea* spore suspension (2 × 10^5^ spores/ml) and photos were taken at 5 dpi. **(B)** Chlorophyll contents in leaves of Col-0, AtERF15-OE and AtERF15-Ri plants after *B. cinerea* infection. Chlorophyll was extracted from entire rosette leaves of six mock-inoculated and six *B. cinerea*-inoculated plants for each line and chlorophyll content is shown as percentages of that in the corresponding mock-inoculated plants. **(C)** Percentages of died plants of Col-0, AtERF15-OE and AtERF15-Ri lines after *B. cinerea* infection. Each experiment contained at least two plants per line and plants were scored as died when they were completely rotten. **(D)** *In planta* growth of *B. cinerea* in the Col-0, AtERF15-OE and AtERF15-Ri plants. Measurement of fungal growth was carried out by simultaneous quantification of the transcript levels of *B. cinerea BcActinA* gene and an *Arabidopsis AtActin* gene and relative fungal growth was shown as ratios of the transcript levels of *BcActinA*/*AtActin*. Data presented are the means ±*SD* from three independent experiments and different letters above the columns indicate significant differences at *p* < 0.05 level between the AtERF15-OE or AtERF15-Ri and Col-0 plants.

We next explored the possible role of AtERF15 in immunity against *B. cinerea*, a typical necrotrophic fungal pathogen with distinct infection style from that of *Pst* DC3000, by comparing the disease phenotype of the AtERF15-OE and AtERF15-Ri plants. In the Col-0 plants, typical necrotic and chlorotic lesions of disease symptom were seen at 4 dpi; however, these lesions were not merged into large necrotic area during the 8-day experimental period (**Figure 5A**). Relative chlorophyll contents in leaves of the inoculated plants were reduced by ∼18% at 6 dpi (**Figure 5B**) and ∼14% of the inoculated plants were completely died at 12 dpi (**Figure 5C**). In contrast, the AtERF15-OE plants displayed less disease symptom and no significant lesion was seen even at 8 dpi (**Figure 5A**). The relative chlorophyll content in leaves of the AtERF15-OE-1 and AtERF15-OE-2 plants was slightly reduced by ∼15% (**Figure 5B**) and only 7% of the inoculated plants were completely died at 12 dpi (**Figure 5C**). However, the AtERF15-Ri plants exhibited severe susceptibility to *B. cinerea*. At 8 dpi, large necrotic and chlorotic lesions appeared on leaves of the AtERF15-Ri-1 and AtERF15-Ri-2 plants, leading to extensive tissue damage (**Figure 5A**). The relative chlorophyll contents in leaves of the AtERF15-Ri-1 and AtERF15-Ri-2 plants were significantly decreased, leading to a reduction of ∼40% (**Figure 5B**), and ∼30% of the inoculated plants were completely died at 12 dpi (**Figure 5C**). Furthermore, *in planta* growth of *B. cinerea* in inoculated plants was also measured to confirm the disease phenotypes observed in AtERF15-OE and AtERF15-Ri plants. To do this, the accumulation of the *B. cinerea* actin A (*BcActinA*) gene transcript, which is indicative of the rate of *in planta* fungal growth (Benito et al., 1998), was determined and compared among the Col-0, AtERF15-OE and AtERF15-Ri plants using qRT-PCR. Accumulation of the *BcActinA* transcript in the Col-0, AtERF15-OE and AtERF15-Ri plants was correlated well with the disease symptoms (**Figure 5D**). In the Col-0 plants, the abundance of the *BcActinA* transcript increased as infection advanced (**Figure 5D**). In the AtERF15-OE plants, the *BcActinA* transcript increased slower and accumulated to significant lower levels, resulting in reduction of ∼50 and ∼65% at 24 and 48 hpi, respectively, as compared to those in the Col-0 plants (**Figure 5D**). In contrast, significantly higher levels of the *BcActinA* transcript accumulated in the AtERF15-Ri plants, leading to increases of ∼90 and ∼105% at 24 and 48 hpi, respectively, as compared to those in the Col-0 plants (**Figure 5D**). Taken together, these data indicate that OE of *AtERF15* led to decreased susceptibility while suppression of *AtERF15* resulted in increased susceptibility to *B. cinerea*, as confirmed by both the disease phenotypes and *in planta* fungal growth.

**FIGURE 6:**
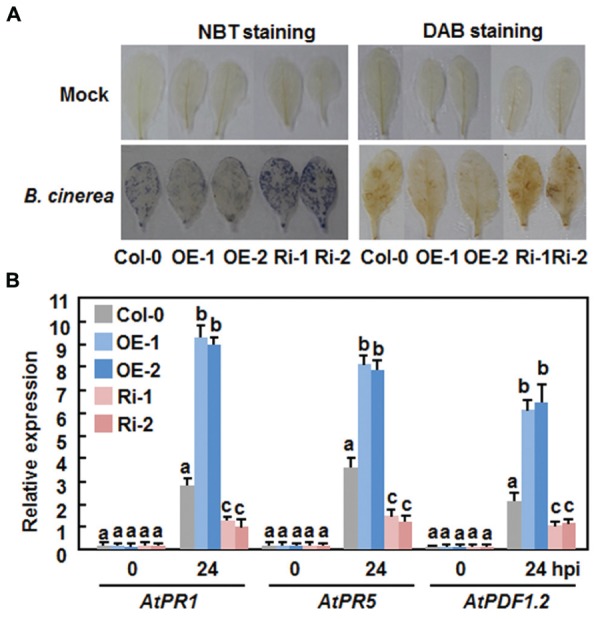
**Altered ROS accumulation and defense gene expression in *AtERF15*-OE and *AtERF15*-Ri plants after infection with *B.cinerea***. Four-week-old plants were inoculated by foliar spraying with *B. cinerea* spore suspension or mock inoculated with similar volume of solution and leaf samples were taken 24 h after inoculation. **(A)** *In situ* detection of accumulation of superoxide anion (left) and H_2_O_2_ (right) after infection of *B. cinerea*. Accumulation of superoxide anion and H_2_O_2_ in leaves was detected by NBT staining and DAB staining, respectively. **(B)** Expression of defense genes after *B. cinerea* infection. Expression of defense genes was analyzed by qRT-PCR and the transcript data were normalized with the value of a reference *AtActin* gene. Relative expression levels of the defense genes were expressed as folds of the *AtActin* gene. Data presented are the means ± *SD* from three independent experiments and different letters above the columns indicate significant differences at *p* < 0.05 level between the AtERF15-OE or AtERF15-Ri and Col-0 plants.

Generation and accumulation of reactive oxygen species (ROS) have been implicated in susceptible responses against necrotrophic fungi including *B. cinerea* (Mengiste, 2012). We thus compared the production of ROS in the Col-0, AtERF15-OE and AtERF15-Ri plants after *B. cinerea* inoculation by *in situ* NTB staining of superoxide anion (Doke, 1983) and DAB staining of H_2_O_2_ (Thordal-Christensen et al., 1997). In mock-inoculated plants, no significant accumulation of superoxide anion and H_2_O_2_ was seen in the Col-0, AtERF15-OE and AtERF15-Ri plants (**Figure 6A**). Upon infection of *B. cinerea*, dramatic accumulation of superoxide anion and H_2_O_2_ was shown in leaves of the Col-0 plants at 48 h after inoculation (**Figure 6A**). When compared with those of the Col-0 plants, however, less accumulation of superoxide anion and H_2_O_2_ was detected in leaves of the AtERF15-OE plants, whereas more accumulation of superoxide anion and H_2_O_2_ was seen in leaves of the AtERF15-Ri plants (**Figure 6A**). These results indicate that OE or suppression of *AtERF15* affected the generation and accumulation of ROS upon infection of *B. cinerea*.

We further compared the expression changes of some defense-related genes in Col-0, AtERF15-OE and AtERF15-Ri plants after infection of *B. cinerea* to explore the relationship between the altered immunity and transcriptional defense response. Without infection of *B. cinerea* (0 hr), the expression levels of these selected defense genes in the AtERF15-OE and AtERF15-Ri plants were comparable to those in the Col-0 plants (**Figure 6B**), indicating that OE or suppression of *AtERF15* did not affect the defense response in plants. Upon infection of *B. cinerea*, the expression of *AtPR1*, *AtPR5*, and *AtPDF1.2* in the Col-0 plant was significantly upregulated, as compared with those in the mock-inoculated plants, at 24 h after inoculation with *Pst* DC3000 (**Figure 6B**). When compared with those in the Col-0 plants, the *B. cinerea*-induced expression of *AtPR1*, *AtPR5*, and *AtPDF1.2* showed threefold increase for *AtPR1* and *AtPDF1.2* and twofold increase for *AtPR5* in the AtERF15-OE plants but decreased to half of that in the AtERF15-Ri plants at 24 h after inoculation (**Figure 6B**). These results suggest the OE or suppression of *AtERF15* can affect the activation of defense response in *Arabidopsis* plants upon infection of *B. cinerea* and the changes in the expression of the selected defense-related genes is correlated to the altered immunity in the AtERF15-OE and AtEF15-Ri plants.

### Attenuation of flg22 and Chitin-Triggered PTI Responses in AtERF15-Ri Plants

**FIGURE 7:**
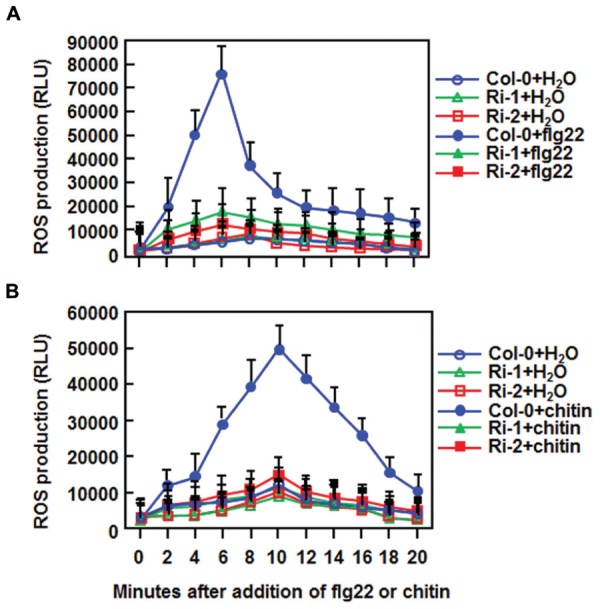
**Abolishment of flg22- and chitin-induced ROS burst in *AtERF15*-Ri plants. (A)** Detection of flg22-induced ROS burst. **(B)** Detection of chitin-induced ROS burst. Leaf samples were harvested from four-week-old plants and ROS burst was measured by a luminol-based assay immediately after addition of flg22 (100 nM), chitin (1 μM) or solution. Repeated experiments showed similar results.

**FIGURE 8:**
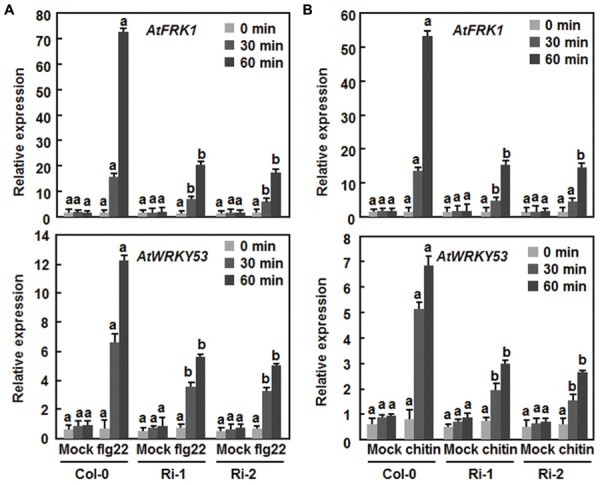
**Attenuation of flg22- and chitin-induced expression of PTI marker genes in *AtERF15*-Ri plants. (A)** Expression of flg22-induced expression of *AtFRK1* and *AtWRKY53*. **(B)** Expression of chitin-induced expression of *AtFRK1* and *AtWRKY53*. Disks from leaves of four-week-old plants were treated with flg22 (100 nM), chitin (1 μM) or solutions as mock controls. Relative expression levels of the defense genes were expressed as folds of the *AtActin* gene. Data presented are the means ± *SD* from three independent experiments and different letters above the columns indicate significant differences at *p* < 0.05 level between the AtERF15-Ri and Col-0 plants at the same time points.

The fact that suppression of *AtERF15* decreased immunity against *Pst* DC3000 (**Figure 4**) led us to hypothesize that AtERF15 may have a function in regulation of PTI response. To test this hypothesis, we first compared the PAMP-induced ROS burst, an early PTI response (Kadota et al., 2014), between the Col-0 and AtERF15-Ri plants after treatment with flg22 or chitin, two typical PAMPs derived from bacteria and fungi, respectively. In these assays, no constitutive ROS burst was detected in mock-treated Col-0 and AtERF15-Ri plants (**Figures 7A,B**). Significant ROS burst in leaves of the Col-0 plants was observed around 6 and 10 min after addition of flg22 and chitin, respectively; however, only a much reduced ROS burst was seen in leaves of the AtERF15-Ri-1 and AtERF15-Ri-2 plants after addition of flg22 and chitin (**Figures 7A,B**). We next examined and compared the changes in expression of *FLG22-INDUCED RECEPTOR-LIKE KINASE 1* (*AtFRK1*) and *AtWRKY53*, two well-known PTI-responsive genes (Asai et al., 2002; Singh et al., 2012), between the Col-0 and AtERF15-Ri plants after treatment with flg22 or chitin. The expression levels of *AtFRK1* and *AtWRKY53* were comparable between the Col-0 and AtERF15-Ri plants in mock controls (**Figures 8A,B**), suggesting that suppression of *AtERF15* expression did not affect the expression of these two PTI-responsive genes. Treatments of the leaf disks with flg22 or chitin significantly upregulated the expression of AtFRK1 and AtWRKY53 in the Col-0 plants, leading to >15 folds for *AtFRK1* and >5 folds for *AtWRKY53*, as compared with those in mock controls (**Figures 8A,B**). However, the flg22- and chitin-upregulated expression of *AtFRK1* and *AtWRKY53* was markedly suppressed in the AtERF5-Ri plants, resulting in 50–75% of reduction as compared to those in the Col-0 plants (**Figures 8A,B**). These results indicate that suppression of *AtERF15* attenuated the flg22- and chitin-induced PTI responses in AtERF15-Ri plants, including ROS burst and expression of PTI-responsive genes.

### Attenuation of SA-Induced Defense Response in AtERF15-Ri Plants

**FIGURE 9:**
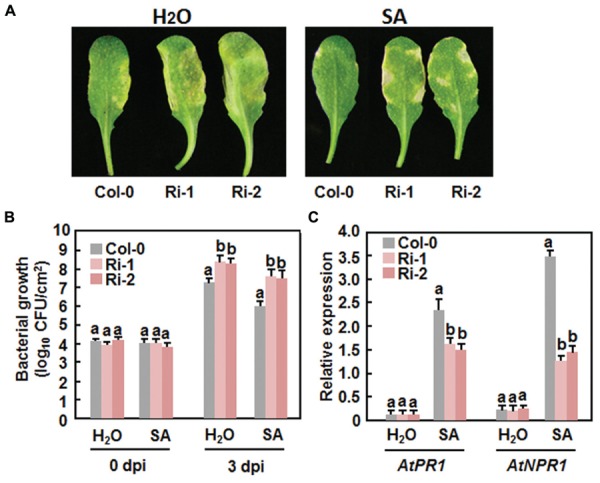
**Attenuation of SA-induced defense response in *AtERF15*-Ri plants. (A)** and **(B)** Attenuated SA-induced resistance against *Pst* DC3000 in the AtERF15-Ri plants. Four-week-old plants were treated by 1 mM SA or water and then infiltration-inoculated with *Pst* DC3000 (OD_600_ = 0.002) 24 hr later. **(A)** Disease symptom in representative inoculated leaves at 4 dpi and **(B)** bacterial populations in inoculated leaves. **(C)** Expression changes of defense genes after SA treatment. Four-week-old Col-0 and AtERF15-Ri plants were treated with 1 mM SA and expression of defense genes was analyzed using qRT-PCR. The transcript data obtained were normalized with the value of a reference *AtActin* gene and relative expression of the defense genes as shown as folds of the *AtActin* gene. Data presented are the means ± *SD* from three independent experiments and and different letters above the columns indicate significant differences at *p* < 0.05 level between the AtERF15-Ri and Col-0 plants.

The fact that the expression of *AtERF15* was upregulated by SA led us to examine whether AtERF15 is required for SA-induced defense response. To test this hypothesis, we first assessed the SA-induced disease resistance in the AtERF15-Ri plants by comparing disease phenotypes and bacterial growth in Col-0 and AtERF15-Ri plants after *Pst* DC3000 infection. The SA-treated Col-0 plants showed less severe disease symptom than the water-treated plants at 4 dpi (**Figure 9A**). The SA-treated AtERF15-Ri plants showed less disease than the water-treated plants but displayed more disease than the SA-treated Col-0 plants (**Figure 9A**). At 3 dpi, the bacterial growth was calculated as 1.4 × 10^7^ CFU/cm^2^ in water-treated Col-0 plants and (2.1 and 2.6) × 10^8^ CFU/cm^2^ in water-treated AtERF15-Ri plants, and as 1.1 × 10^6^ CFU/cm^2^ in SA-treated Col-0 plants and (3.2 and 4.4) × 10^7^ CFU/cm^2^ in SA-treated AtERF15-Ri plants, respectively (**Figure 9B**). The bacterial population in SA-treated Col-0 plants was reduced by 10.4 folds, whereas the bacterial populations in the SA-treated AtERF-Ri-1 and AtERF-Ri-2 plants were decreased by 6.5 and 5.9 folds, respectively, compared with those in water-treated Col-o and AtERF15-Ri plants (**Figure 9B**). We further compared the expression changes of *AtPR1*, a marker defense gene in SAR, and *AtNPR1*, a key regulatory gene critical for SAR, in Col-0 and AtERF15-Ri plants after SA treatment. In the Col-0 plants, SA significantly induced expression of *AtPR1* and *AtNPR1* at 24 h after treatment (**Figure 9C**). Although SA also induced expression of *AtPR1* and *AtNPR1* in the AtERF15-Ri plants, their expression levels were significantly reduced, leading to reduction of 40% for *AtPR1* and 120% for *AtNPR1*, as compared with those in the Col-0 plants (**Figure 9C**), indicating an attenuated SA-induced defense response in AtERF15-Ri plants. Collectively, these results demonstrate that suppression of AtERF15 partially impaired the SA-induced defense responses in AtERF15-Ri plants.

## Discussion

The importance of the B3 group ERFs in plant immunity has been documented by the characterization of seven members as positive or negative regulators of immune responses (Berrocal-Lobo et al., 2002; Lorenzo et al., 2003; Oñate-Sánchez et al., 2007; Pré et al., 2008; Moffat et al., 2012). In the present study, OE of *AtERF15* conferred increased resistance while RNAi-mediated suppression of *AtERF15* led to decreased resistance against *Pst* DC3000 and *B. cinerea* (**Figures 4** and **5**). Furthermore, RNAi-mediated suppression of *AtERF15* also attenuated flg22- and chitin-induced PTI response (**Figure 7**) and partially impaired the SA-induced defense response (**Figure 8**). These findings demonstrate that AtERF15 acts as a positive regulator of *Arabidopsis* immune responses against *Pst* DC3000 and *B. cinerea*, representing a hemibiotrophic bacterial pathogen and a necrotrophic fungal pathogen, respectively.

Generally, defense responses against (hemi)biotrophic pathogens such as *Pst* DC3000 are modulated via the SA signaling pathway, while defense responses against necrotrophic pathogens like *B. cinerea* are thought to be mediated by the JA/ET signaling pathways (Glazebrook, 2005; Grant and Jones, 2009; Verhage et al., 2010). Whereas most of the studies reported antagonistic interaction between the SA and JA/ET signaling pathways, positive cross-talks between these two defense signaling pathways do exist in response to pathogen infections (Glazebrook, 2005; Mur et al., 2006; Derksen et al., 2013). Previous studies have shown that AtERF1, AtERF5, AtERF6, AtERF14, and ORA59 in the B3 group act as regulators of the JA/ET signaling pathway (Berrocal-Lobo et al., 2002; Lorenzo et al., 2003; Oñate-Sánchez et al., 2007; Pré et al., 2008; Moffat et al., 2012). However, our data presented in this study imply that AtERF15 may function in both of the SA and JA/ET signaling pathways. Firstly, expression of *AtERF15* was induced by *Pst* DC3000 and *B. cinerea* as well as by SA and MeJA (**Figure 1**). ET also induced expression of *AtERF15* (Oñate-Sánchez et al., 2007). Secondly, OE of *AtERF15* led to increased resistance while suppression of *AtERF15* resulted in decreased resistance against *Pst* DC3000 and *B. cinerea* (**Figures 4** and **5**). This is different from the functions of AtERF5 and AtERF6, which have positive functions in defense against *B. cinerea* but negatively regulate defense against *Pst* DC3000 (Moffat et al., 2012). The function of AtERF15 in defense against *B. cinerea* is supported by the patterns of pathogen-induced ROS accumulation in AtERF15-OE and AtERF15-Ri plants, which correlates with the general concept that excessive accumulation of ROS often benefits the infection by *B. cinerea* (Mengiste, 2012). Thirdly, expression of both SA- and JA-responsive defense genes was affected with similar patterns in AtERF15-OE and AtERF15-Ri plants by infection of *Pst* DC3000 and *B. cinerea* (**Figures 4C and 6B**). It was observed that OE of some B3 group members can constitutively upregulate the expression levels of some defense-related genes (Oñate-Sánchez et al., 2007; Pré et al., 2008; Moffat et al., 2012). By contrast, OE or suppression of *AtERF15* had no effect on the expression of the defense genes examined (**Figures 4** and **6**). The expression levels of the defense genes exhibited significant additional increases in the AtERF15-OE plants but were decreased in the AtERF15-Ri plants, as compared with those in the Col-0 plants, after pathogen infection (**Figures 4** and **6**). These observations indicate that AtERF15 functions as a positive regulator to prime the defense response upon pathogen infection. Furthermore, the involvement of AtERF15 in the SA signaling pathway can be further validated by the facts that the SA-induced defense response in the AtERF15-Ri plants was partially impaired (**Figure 9**). The observations that SA induced expression of *AtERF15* (**Figure 1**) and the SA-induced expression of *AtNPR1* in AtERF15-Ri plants was significantly suppressed (**Figure 9**) imply that AtERF15 may function between SA and NPR1 in the SA signaling pathway. Further analyzing the AtERF15-dependent expression patterns of *AtICS1* and *AtWRKY33* in AtERF15-Ri plants will clarify the mechanism whether the attenuated SA-dependent defense responses in AtERF15-Ri plants is caused by the effect of *AtERF15* on the SA biosynthesis or on the later signaling. Lastly, the abolishment the flg22- and chitin-induced ROS burst (**Figure 7**) and the expression of PTI marker genes *AtFRK1* and *AtWRKY53* (**Figure 8**) in AtERF15-Ri plants not only demonstrates the requirement of AtERF15 in innate immune response but also further supports the conclusion that AtERF15 is a positive regulator of defense responses against *Pst* DC3000 and *B. cinerea*. However, the specific involvement of AtERF15 in PTI sub-branches needs to be further studied. AtERF5 was found to negatively regulate chitin signaling and defense response to *Alternaria brassicicola* but positively regulate SA signaling and defense response to *Pst* DC3000 (Son et al., 2012). Therefore, it appears that AtERF15 and AtERF5 play roles in innate immunity response with different mechanisms, although they belong to the same B3 group.

It was previously reported that AtERF14 plays a non-redundant role in defense response against *Fusarium oxysporum* (Oñate-Sánchez et al., 2007). In the present study, OE or RNAi-mediated suppression of *AtERF15* led to clear phenotype alterations in disease resistance against *Pst* DC3000 and *B. cinerea* (**Figures 4** and **5**). Thus, AtERF15 and AtERF14, two closely related members in the B3 group (Nakano et al., 2006), function independently in *Arabidopsis* immune responses. However, *AtERF15* showed reduced induction by ET in *aterf14* mutant plants (Oñate-Sánchez et al., 2007), indicating a functional relationship between AtERF14 and AtERF15 in defense response.

The involvement of the ERF proteins in plant growth and development has been well-documented (Licausi et al., 2013). Among the three group members, OE of *AtERF1*, *ORA59* or *AtERF14* was found to cause significant growth inhibition phenotypes under normal conditions and upregulated expression of defense genes (Solano et al., 1998; Oñate-Sánchez et al., 2007; Pré et al., 2008). By contrast, we did not observed any morphological and developmental changes (**Figure 3**) and altered expression of defense genes (**Figures 4** and **6**) in the AtERF15-OE and AtERF15-Ri plants grown under normal condition. When overexpressed in Ws-0 background, the mature AtERF15-OX plants were morphologically similar to wild-type plants (Lee et al., 2015). Similar observations were also obtained for *AtERF5* and *AtERF6*, whose OE and T-DNA insertion mutant plants are morphologically normal as the wild type plants (Son et al., 2012). Thus, it is likely that AtERF15 has limited function in growth and development.

Together with the recent finding that AtERF15 functions as a positive regulator of ABA response (Lee et al., 2015), it can be concluded that AtERF15 is a critical regulator of biotic and abiotic stress responses in *Arabidopsis*. Our biochemical assays revealed that AtERF15 is a nucleus-localized transcriptional activator (**Figure 2**) (Lee et al., 2015). It is likely that AtERF15 regulates directly or indirectly the expression of downstream target genes. This is partially supported by the observations on the altered expression patterns of some selected defense-related genes in AtERF15-OE and AtERF15-Ri plants after pathogen infection (**Figures 4** and **6**). On this regard, identification of downstream target genes and their functions will be helpful to elucidate the mechanism for the action of AtERF15 in regulation of biotic and abiotic stress responses in *Arabidopsis*. Furthermore, it is worth to point out that the timely dynamics of AtERF15 function seems to play roles in different aspects of immune response because AtERF15 is not only required for oxidative burst in PTI but also is involved in hormone signaling pathways, which occur later in PTI. This suggests that the AtERF15 protein can be differentially or effectively activated upon elicitation and pathogen infection.

## Author Contributions

HZ, LH, YD, SL, YH, LT, LhH, ZC, and DL carried out most of the experiments. FS and HZ designed the experiments and wrote the paper. All authors read and approved the final manuscript.

## Conflict of Interest Statement

The authors declare that the research was conducted in the absence of any commercial or financial relationships that could be construed as a potential conflict of interest.
